# Microsporidian parasite impairs colony fitness in bumblebees

**DOI:** 10.1098/rsob.240304

**Published:** 2025-02-26

**Authors:** Domenic W. Camenzind, Selina Bruckner, Peter Neumann, Annette Van Oystaeyen, Verena Strobl, Geoffrey R. Williams, Lars Straub

**Affiliations:** ^1^ Institute of Bee Health, Vetsuisse Faculty, University of Bern, Bern, Switzerland; ^2^ Department of Entomology and Plant Pathology, Auburn University, Auburn, AL, USA; ^3^ Agroscope, Swiss Bee Research Centre, Bern, Switzerland; ^4^ Biobest Group NV, Westerlo, Belgium; ^5^ Department of Biological Sciences, Centre for Ecology, Evolution and Behaviour, Royal Holloway University of London, Egham, UK

**Keywords:** *Bombus*, conservation, hypopharyngeal glands, sperm, spillover

## Abstract

Emerging infectious diseases can have a major impact on fitness of novel hosts, thereby contributing to ongoing species declines. In social insects, collaborative brood care by workers and successful mating of male sexuals are key to colony fitness. The microsporidian endoparasite *Nosema ceranae* has spread almost globally, shifting across honeybee species and now to bumblebees. However, despite *N. ceranae* being linked to recent population declines, its possible impact on bumblebee colony fitness remains poorly understood. Here, we show that *N. ceranae* infections can significantly impact *Bombus terrestris* worker feeding glands, as well as longevity, sperm quality and mating abilities of drones. In the laboratory, workers and drones were either exposed to the parasite or not. Then, parasite infection rates and loads, as well as lethal and sublethal parameters, were assessed. Infected drones revealed higher parasite infection rates and spore titres, as well as mortality compared with female workers, suggesting sex-specific susceptibility. Furthermore, infections impaired feeding glands, affected sperm traits and altered mating behaviour, all of which are key to colony fitness. Our findings provide a mechanistic explanation on how *N. ceranae* contributes to the ongoing decline of wild bumblebee populations, calling for respective mitigation measures.

**Figure:**
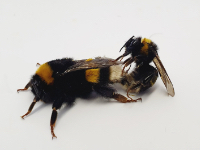

## 1. Introduction

Eusocial insects, including ants, termites, wasps and bees, are among the most ecologically successful and abundant organisms on Earth. By playing a critical role in processes such as pollination, decomposition and predation, they are crucial for maintaining ecological balance and productivity [1]. A colony of social insects can range between a few dozen to millions of individuals that collectively function as a superorganism, and it is resilient to high losses of colony members—as long as the germ line and functionality of the colony are maintained [2,3]. However, there is substantial evidence suggesting widespread declines and losses of wild and managed eusocial insects, raising considerable concern due to the anticipated negative impacts on environmental integrity, human health and economic repercussions [4,5]. Understanding the underlying mechanisms responsible for the observed vulnerability of eusocial insects therefore appears key to mitigate the ongoing losses and declines.

Regardless of the stressor’s nature, any reduction in fitness will have profound consequences for a species. In essence, eusocial insect fitness is defined by the number of successfully mated male sexuals and female gynes (i.e. future queens) that establish new colonies [6]. Due to the complex social structure of eusocial insects (i.e. reproductive division of labour, overlapping generations and collaborative brood care [6]), measuring colony fitness can be inherently difficult. For instance, the worker caste typically gains inclusive fitness by ensuring colony functionality through foraging, thermoregulation and brood care, which are critical for colony development and the production of male and female sexuals. However, evidence indicating that impaired bumblebee worker physiology critical for optimal brood care, specifically the hypopharyngeal glands (HPGs), are currently sparse. Although the function of bumblebee HPGs are not fully understood, they have been proposed to be important for digestion [7], endocrine regulation during nutritional uptake [8] and storing vitellogenin [9]. Therefore, these glands are likely to be essential during collaborative brood care, a key element of eusociality and so indirectly affect colony reproduction and fitness. From the male perspective, fitness is primarily defined by their reproductive success (i.e. the number of offspring sired), and thus their ability to mate, as well as the quantity and quality of sperm, are critical [10]. However, to date, there are few data investigating the effects of environmental stressors on male bumblebee reproductive physiological and mating behavioural.

Bumblebees are an ideal model organism to study the consequences of environmental stressors on fitness, as they can easily be kept under laboratory as well as (semi-)field conditions. Various ecotoxicological studies have revealed that inadvertent pesticide exposure can reduce colony development and the number of sexuals produced, as well as negatively affect wild bee population dynamics [11]. These effects are likely explained by pesticides hampering key tokens of fitness such as survival [12], foraging abilities [13], feeding gland development [14], nursing behaviour and thermoregulation [15], as well as sperm traits [16] and mating behaviour [17]. While there are data showing negative effects of parasites on bumblebee colony growth and production of sexuals [18], effects of parasites and pathogens on key tokens of bumblebee fitness remain scarce.

Social bees are known to be infected by a vast array of parasites and pathogens [19]. Recently, much attention has been devoted to the threat of emerging infectious diseases across insect pollinators [20]. Indeed, the microsporidian parasite *Nosema ceranae* has emerged as a significant disease in honeybees (*Apis* spp.). Originally a parasite of the eastern honeybee *Apis cerana*, endemic to Asia, *N. ceranae* has spread across the genus *Apis*, including western honeybees *Apis mellifera*, and has now rapidly spread globally [21]. In honeybees, the parasite is known to negatively impact adult survival, immunocompetence, feeding behaviour and physiological development [22–25] and has been argued to impair colony health [26]. Given the parasite is mainly transmitted through the faecal-oral route, and excreted spores can persist for long periods of time in the environment (e.g. flowers) [27], it is no surprise that there is an increasing body of molecular evidence suggesting *N. ceranae* can be found in bumblebees (e.g. [28])—suggesting a pathogen spillover occurring. Yet, despite the rapid geographical expansion of *N. ceranae* and the increasing reports of native bumblebee species across Europe and the Americas being infected with the parasite, our understanding of the potential implications on bumblebee health remain scarce. The available data show that *N. ceranae* can reduce survival as well as alter feeding behaviour and impair learning abilities in bumblebees [29–31]. While these effects undoubtedly will render negative effects at the individual and likely also at the colony level, identifying potential effects on key physiological and behavioural traits that will directly reduce bumblebee colony fitness are lacking.

To explore potential mechanistic explanations for reports of reduced colony fitness and population declines due to pathogen spillovers, we investigated the lethal and sublethal effects of *N. ceranae* exposure on individual adult *Bombus terrestris* worker and drones (= males). Therefore, under controlled laboratory conditions, workers and drones were exposed to the parasite or not. Then, lethal (i.e. survival) as well as sublethal effects (i.e. parasite infection rates and loads, food consumption, feeding gland development and drone mating behaviour as well as sperm traits) were assessed to determine the impact of parasite exposure on key fitness traits as well as to compare the virulence of the parasite between workers and drones. Considering previous studies [30,32], we hypothesized that *N. ceranae* would impair behaviour, tissue and organ development, as well as reproductive physiology in workers and drones. Furthermore, we expected the degree of susceptibility to be increased in haploid drones due to hemizygosity at immune loci, probably reducing tolerance to infections [33].

## 2. Material and methods

The experiment was conducted between May and September 2019 at the Institute of Bee Health, University of Bern, Switzerland. To establish known age-cohorts of bees, newly emerged callow *B. terrestris* workers and drones (*n* = 280) were selected at Biobest Group NV, Westerlo, Belgium. Callow bumblebees can easily be identified by the lack of hair pigmentation during the first 24 h after emergence [34]. Due to shipping to Switzerland, the bees were between two and 3 days of age upon arrival. Visual inspections revealed that all individuals were free of obvious physical abnormalities, clinical symptoms of disease or ectoparasite infestations. Nevertheless, 30 bees (*N*_workers_ = 15; *N*_drones_ = 15) were randomly selected and analysed using standardized PCR methods to determine potential *a priori* parasite infestations (e.g. *Nosema bombi*, *N. ceranae* or *Crithidia bombi*) following [35–37]. All specimens tested revealed negative results.

### 2.1. *Nosema ceranae* propagation and inoculation

Following [30], *N. ceranae* spore solutions were freshly obtained from honeybee (*Apis mellifera* L.) workers originating from local colonies and *N. ceranae* was confirmed using standard qPCR protocols, including species-specific PCR primers [37]. In brief, to obtain spore solutions of known concentrations, five *N. ceranae* positive foragers (all negative for *N. apis*) were used to infect newly emerged caged honeybee workers through bulk feeding [36]. In parallel, cages of control non-infested honeybee workers were maintained and kept together with the *N. ceranae* infected bees in an incubator over 12 days, during which bee survival was monitored daily. Then, three random midguts of either *N. ceranae* infested or control workers were pooled in a separate vial with 1.0 ml distilled water. After homogenization, each vial solution was checked under the light microscope (×400 objective) for the presence of *N. ceranae* spores [36]. The *N. ceranae* spore load in the positive sample was quantified using a haemocytometer and light microscopy (Thermo Fischer Scientific, Waltham, Massachusetts, USA) [38]. Prior to the treatment, the initial body mass (hereafter start mass) of each bumblebee (*N*_drones_ = 124; *N*_workers_ = 124) was recorded to the nearest 0.1 mg using an analytic scale (Mettler Toledo AT400). Then, all bees were starved for 2 h in individual cages. Afterwards, bees were randomly assigned to either the parasite or control treatment. Bees were individually hand-fed a sucrose solution with either *N. ceranae* spores (10 000 spores per individual in 5 µl 50% (w/w) sucrose solution) or free of *N. ceranae* spores (5 µl 50% (w/w) sucrose solution). To ensure that each specimen ingested the sucrose solution, the pipette tip was held near the antennae of each individual to trigger the proboscis extension reflex, thereby stimulating feeding behaviour [39].

### 2.2. Consumption, survival and spore counts

Bees were maintained individually in standard cages (100 cm^3^) and maintained in incubators at 28°C and 60% relative humidity (RH) in complete darkness [14]. Each bee was provided with 50% [w/w] sucrose solution *ad libitum* through a 5 ml syringe to provide sufficient carbohydrates. Syringes were weighed and exchanged every 4 days to measure consumption as well as to prevent possible fungal growth. Sucrose consumption was measured by recording the mass of the syringe every 4 days until the experiment was terminated or at the point of bee death. Furthermore, to ensure each bee had ample protein resources for organ and tissue development, approximately 1 g of pesticide-free corbicular honeybee pollen—formed in a small ball—was provided in each cage. Pollen consumption was measured by recording the mass of the provided pollen ball at either day 12 or the point of death following [14]. In parallel, three cages without a bee but with a sucrose feeder and a pollen ball were included to measure the evaporation rate, which was then accounted for in the consumption measurements. Furthermore, the consumption measures were divided by an individual’s start mass to obtain relative consumption values. We determined the relative daily sucrose consumption (hereafter daily sucrose consumption; g g^−1^ bee^−1^ day^−1^), the total relative consumption (hereafter total consumption; g g^−1^ bee^−1^) as well as the total relative pollen consumption (hereafter pollen consumption; g g^−1^ bee^−1^) and thus correcting for bodyweight when comparing consumption rates. Survival was recorded daily. *Nosema ceranae* spore counts were determined for all individuals (*N*_drones_ = 96; *N*_workers_ = 109) that survived 12 days post-treatment exposure—sufficient time for the parasite to replicate [40]. If *N. ceranae* spores could be observed, an infection was diagnosed. Spore counts were not conducted in deceased specimen due to postmortem-desiccation and gut tissue autolysis.

### 2.3. Hypopharyngeal gland assessment

A subset of workers surviving 12 days (i.e. age 15) were used (*N*_per treatment_ = 32). While the HPG acini size in bumblebees can remain consistent until day 50 depending on the bees colony task allocation, we chose day 15 to possibly enable comparisons with previous studies using honeybees, where maximum acini size is reached between 8 and 15 days [41] and bumblebees [14]. To measure the HPG acini size, workers were decapitated after anaesthetization with CO_2_ for 20 seconds; heads were stored separately in 2 ml Eppendorf tubes containing 0.5 ml of 2% paraformaldehyde PBS preservation buffer [42], and kept at 4°C until the HPGs were dissected. In brief, each head was air-dried for 5 min after removal from their tube. Once dry, the head was glued at the posterior end on a wooden dissection block; antennae were then removed at their junctions and the frons (frontal head skin) was lifted off after a cut was made across the ocelli, along the margins of the compound eyes and the mask, but excluding clypeus, labrum and mandibles. The exposed HPGs were then removed using forceps and deposited in a Petri dish containing 0.5 ml saline dissection buffer for 5 min. Single acini were accentuated by adding 0.5 ml of Coomassie Brilliant Blue g−250 stain, which was dispensed throughout the extracted glands by gently shaking the Petri dish. After 5 min, the glands were mounted on a wetted glass slide using a coverslip. Slide mounted acini were examined using a 5× compound light microscopy (Olympus BX41) and digital microscope photography (Olympus DP72). Thirty acini diameters per individual were measured perpendicular to their attachment point with the imaging software ImageJ 1× using a 50 µm ruler as a measurement scale [43].

### 2.4. Sperm assessment

Drones surviving 12 days (*n* = 97) were assessed for sperm quantity and sperm viability following [17]. All drones were considered sexually mature, as they commonly begin mating at the age of 4 days onwards [44]. Drones were briefly anaesthetized using CO_2_ before being pinned to a wax plate and dissected. Sperm samples were collected by removing the entire genitalia, including the testis, accessory gland, granular gland, vesicula seminalis and ejaculatory duct from each drone; these were placed in a 1.5 ml Eppendorf tube containing 200 µl Kiev^+^ buffer and gently crushed to form a diluted sperm stock solution. Immediately after, a 50 µl aliquot of the sperm stock solution was set aside in a separate 1.5 ml Eppendorf tube for analyses of sperm viability (proportion of sperm alive). Sperm viability was quantified according to [45]. In brief, each sample was diluted with 50 µl of Kiev^+^ buffer before 1 µl of propidium iodide (PI) solution (1 mg ml^− 1^) and 0.5 µl of Hoechst 33 342 (0.5 mg ml^− 1^) (both Sigma-Aldrich) were added to the suspension. Samples were then incubated for approximately 20 min in complete darkness and then gently vortexed. Ten microlitres were viewed at 400× magnification using fluorescent microscopy (Olympus BX41, Switzerland) equipped with filter cubes for UV excitation. Ten visual fields were randomly selected for each sample to quantify living and dead sperm, and an average value was then calculated upon these fields. Sperm counts were performed by adding 50 µl of stock sperm solution diluted in 50 µl Kiev^+^ buffer (1:1 dilution) in a 1.5 ml Eppendorf tube. Sperm quantity was then measured using a Neubauer counting chamber under light microscopy (Thermo Fischer Scientific, USA) and quantified by applying the following calculation: Total sperm quantity (200 µl) = average number of sperm counted in two Neubauer counting chambers × dilution factor (1:1 dilution) × sperm volume used for Neubauer counting chamber (10 µl) × stock solution volume (200 µl). Then, the total sperm count was multiplied with the percentage of living sperm to receive the total living sperm.

### 2.5. Mating behaviour

Newly emerged virgin bumblebee gynes (*n* = 45) were obtained from the mass rearing of Biobest Group NV (Belgium) and sent in a transparent plastic box (15 × 15 × 10 cm) to Switzerland. The gynes received *ad libitum* access to pesticide-free sterilized corbicular honeybee pollen and 50% (w/w) sucrose solution until they were individually placed into a mating arena (250 cm^3^). Then, a subset of 12 day old virgin drones from the control (*n* = 25) and parasite treatment (*n* = 13) was randomly selected and paired with a virgin gyne that was between 8 and 9 days of age as, in captivity, virgin gynes are the most receptive when younger than 11 days [46]. The bees could interact freely within the mating arena. Both mating latency (i.e. the time between the introduction of the drone into the mating arena until initiation of copulation with the gyne) and the duration of mating (i.e. time between copulation initiation to termination) were recorded [17]. If drones did not start mating after 60 min, the mating was considered as unsuccessful; the trial was then aborted, and the pair was separated. The unsuccessful drones were granted a second opportunity to mate, and therefore placed in a new mating arena with a new virgin gyne, to confirm the inability of the drone to initiate mating. Again, mating latency and duration as well as mating success were recorded. Immediately after the mating, all drones (regardless of their mating success) were dissected and the sperm traits (i.e. quantity and quality) of each individual were determined (see §2.4.).

### 2.6. Statistical analyses

All statistical analyses were performed using STATA16, whereas figures were created using NCSS20. The Shapiro–Wilk’s test and the Levene’s test were used to determine the distribution of the data and assess model residues and the homogeneity of variances. Accordingly, the appropriate statistical tests were chosen. To assess the relationship between the explanatory variable (i.e. parasite exposed) and the dependent variables (i.e. total living sperm and HPG-size) linear regression models were applied using the function *regress*, where individual bees were considered independent units and start mass was included as covariate. Additionally, to determine differences between treatments, generalized logistic or linear (regression) models (GLMs) and general linear models (GLM) with random intercepts were fit using the functions *melogit*, *meglm* and g*lm*. Individual bees were considered independent units; parasite exposure, sex and the interaction term (i.e. parasite × sex) were included as the explanatory (fixed) terms; *N. ceranae* spore levels as well as start mass were included as covariates. For each model, a stepwise backward elimination approach was applied to determine the model of best fit. Best fit models were chosen by comparing every multi-level model with its single-level model counterpart with a likelihood ratio (LR) test and comparing different models with the Akaike information criterion (AIC) using the functions *lrtest* and *estat ic*, respectively. Whenever one of the fixed terms revealed significant, Bonferroni multiple-pairwise comparison tests (*bmct*) were performed using the function *mcompare(bonferron*i) [47]. If sex-specific differences were revealed, drones and workers were separated to facilitate analyses. Whenever appropriate, the means ± s.e. or medians ± 95% confidence intervals (CI) are given in the text of the result section.

To account for differences in start mass and to enable sex-specific comparisons, consumption and spore counts were analysed using the relative values. Based on the analysis of residuals, the variables start mass (g), consumption (g g^−1^ bee^−1^), spore counts (spore bee^− 1^), total living sperm (thousands) and HPG diameter (µm), were modelled using a GLMM with either a Gaussian, Gamma or Poisson distribution (electronic supplementary material, table S1). Counter transforming the outcome variables if non-parametrically distributed, we opted for using Gamma distribution that provided good fits (i.e. normality of the residuals) [45]. Survival times for individuals were fitted using the function *mestreg* for multilevel survival models with a Weibull distribution and displayed using Kaplan–Meier survival curves. Survival was calculated by using cumulative survival rates [%] 12 days post-experiment initiation. Data distributions, and the applied models including fixed and random effects used to test for treatment effects of each variable can be found in the electronic supplementary material, table S1.

## 3. Results

### 3.1. Sucrose and pollen consumption

**Figure 1.**
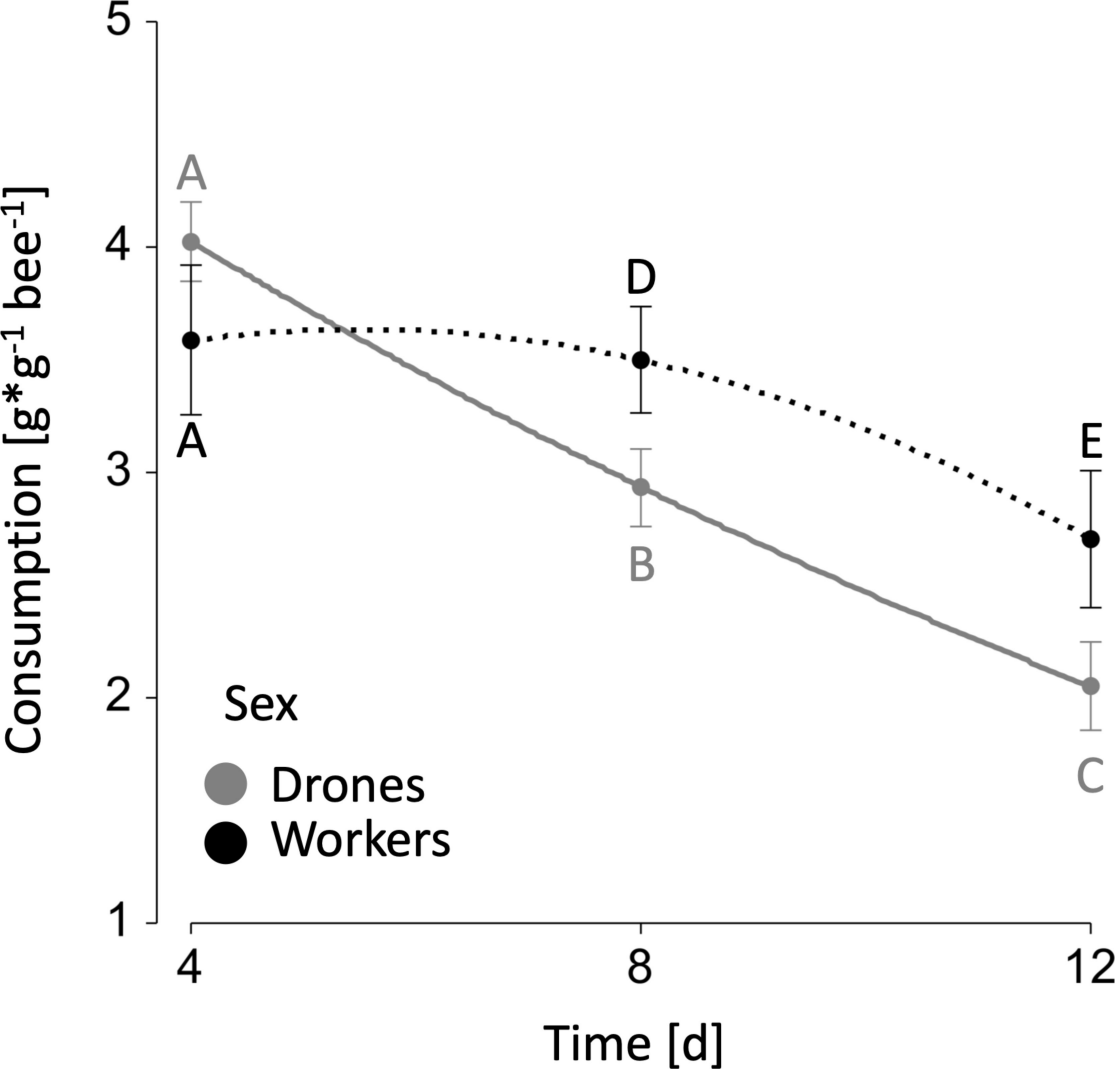
Daily relative consumption over time for male (drone) and worker bumblebees, *Bombus terrestris*: mean daily consumption of sucrose solution, measured at timepoints 4, 8 and 12. The grey line represents drones and the black line workers. While there was no significant difference in consumption on day 4 (*p* > 0.05), workers consumed significantly more sucrose solution than drones on days 8 and 12 (both *p*’s < 0.05). Regardless of sex, bees consumed significantly less sucrose solution with increasing age (*p* < 0.05). Different letters (A–E) indicate significant differences in daily consumption. The whiskers represent means and s.e.

Regardless of sex and exposure, a significant negative correlation was observed between age and daily sucrose consumption (*z* = −10.69, *p* < 0.001), where bees consumed less sucrose solution with increasing age (figure 1). Parasite exposure revealed no significant effect on total sucrose consumption (*z* = 1.70, *p* > 0.05). In contrast, a sex-specific difference in total sucrose consumption was observed (*z* = 2.04, *p* = 0.04), where workers (10.10 ± 2.89) consumed 9% more than drones (9.05 ± 2.20) (mean [g g^− 1^ bee^− 1^] ± s.e.). This difference in total sucrose consumption however only became apparent in the daily sucrose consumption between days 4 and 12 (both *z*’s < − 3.00, *p’s* < 0.041), whereas during the first 4 days of feeding no significant difference was observed (*z* = 2.04, *p* = 0.62; figure 1). Likewise, pollen consumption in drones (0.25 ± 0.02) was significantly lower compared with workers (0.65 ± 0.03) after 12 days (*z* = 4.62, *p* < 0.001; mean [g g^− 1^ bee^− 1^] ± s.e.; electronic supplementary material, figure S1), resulting in a approximately 61% difference in consumed pollen between sexes. Furthermore, parasite exposure revealed no significant negative effect on the pollen consumption of workers (*z* = 1.89, *p* = 0.36), yet it did significantly decrease pollen consumption in drones (*z* = −5.42, *p* < 0.001; electronic supplementary material, figure S1).

### 3.2. Cumulative survival, infection rates and spore counts

**Figure 2.**
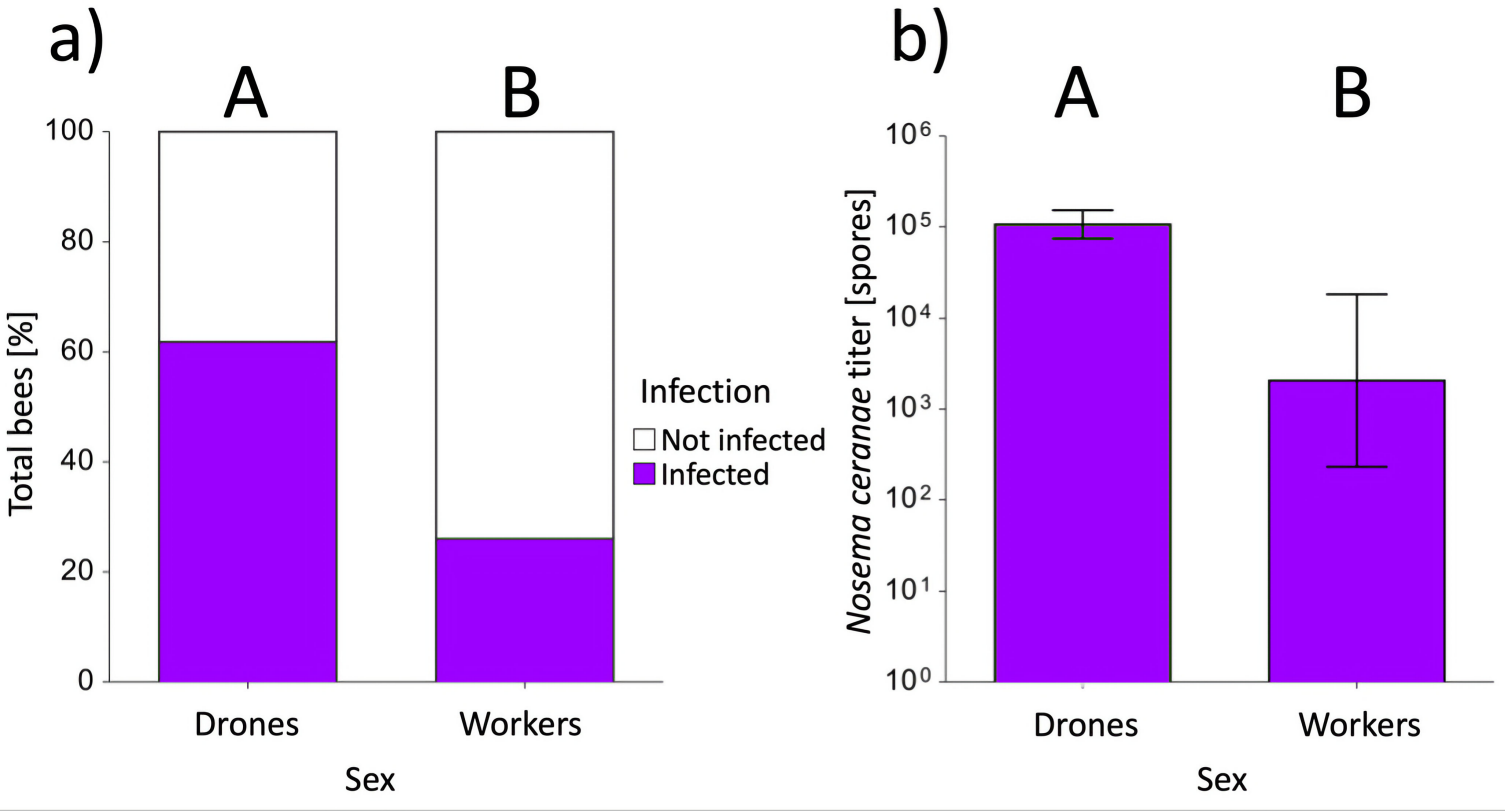
*Nosema ceranae* infection rates and spore titres in infected male (drone) and worker bumblebees, *Bombus terrestris*. (a) Infection rate of exposed bees by sex. The percentage of infected bees is represented in purple, while bees that were not infected are represented in white. Drones had a significantly higher infection rate compared with workers (*p* < 0.05). (b) Mean spore count of infected individuals by sex. Drones had a significantly higher mean spore count than workers (*p* < 0.05). The whiskers represent means and standard errors. In both graphs, capital letters (A, B) indicate significant differences.

Parasite exposure had a significant negative effect on survival (*z* = 2.60, *p* < 0.01), whereas no sex-dependent effect on survival was found (*z* = 1.92, *p* > 0.05). However, a significant interaction between parasite exposure and sex was revealed (*z* < 2.31, *p* < 0.05), whereby control drones (90.32 ± 3.75) had a higher survival rate compared with parasite-exposed drones (70.97 ± 5.76; mean [%] ± s.e.; *z* = 2.85, *p* < 0.05; electronic supplementary material, figure S2). This resulted in 22% reduced survival over 12 days between the parasite-exposed and control drones. In contrast, no significant difference in survival was observed between parasite-exposed and control workers (*z* = 0.52, *p* = 1.00; electronic supplementary material, figure S2); where the cumulative survival rate across both treatments was 87%. Worker control and parasite survival did not significantly differ from their respective drone counterparts (all *z*’s < 2.19, all *p*’s > 0.17; electronic supplementary material, figure S2). *Nosema ceranae* was not diagnosed in either worker or drone controls. Within the parasite exposure treatment, regardless of sex, infected bees exhibited a significantly lower total sucrose consumption (*z* = − 2.11, *p* < 0.05) when compared with exposed but non-infected bees. Also, a significant sex-specific effect was revealed for the infection rates of exposed individuals, whereas drones (61.70 ± 7.17) revealed approximately 58% increased infection rate when comparing with workers (25.93 ± 3.260) (*z* = 2.36, *p* = 0.018; mean (%) ± s.e.; figure 2a). Based on the infected individuals, spore counts were significantly lower in workers (20,535.71 ± 4,464.29) compared with drones (534,568.9 ± 191,571.3); spore load in drones was 26.3-fold higher when compared with those of workers (mean ± s.e. [spore × bee^−1^]; figure 2b).

### 3.3. Hypopharyngeal gland acini size

**Figure 3.**
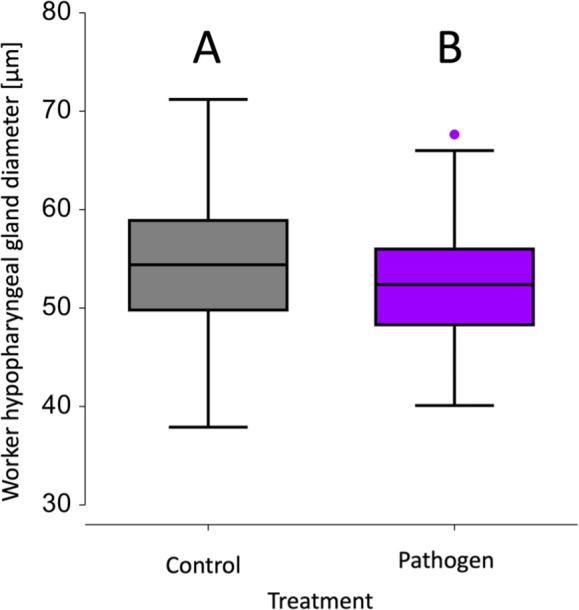
Effect of *Nosema ceranae* infections on HPG acini size of worker bumblebees, *Bombus terrestris*. HPG size of workers exposed with *N. ceranae*, parasite exposed workers (in purple) had a significantly smaller HPG acini size than control workers (in grey) (*p* < 0.05). Alphabetical letters (A, B) indicate significant differences. Additionally, the minimum, the maximum, the sample median and the first and third quartiles are included.

Regardless of the treatment, a significant positive correlation was observed between HPG acini size and worker start mass (*z* = 4.83, *p* < 0.001; electronic supplementary material, figure S3C). Furthermore, both sucrose and pollen consumption had a significant positive effect on HPG acini size (both *z*’s > 1.97, both *p*’s < 0.05; electronic supplementary material, figure S3A,B). However, parasite exposure had a significant negative impact on the HPG acini size (*z* = − 3.95, *p* < 0.001; figure 3); resulting in acini of exposed workers (52.40 ± 0.27) being 3.74% smaller when compared with control acini (54.44 ± 0.26; mean [µm] ± s.e.). No significant correlation was found between spore count and HPG acini size (*t* = − 1.81, *p* > 0.05).

### 3.4. Mating latency, success and duration

**Figure 4.**
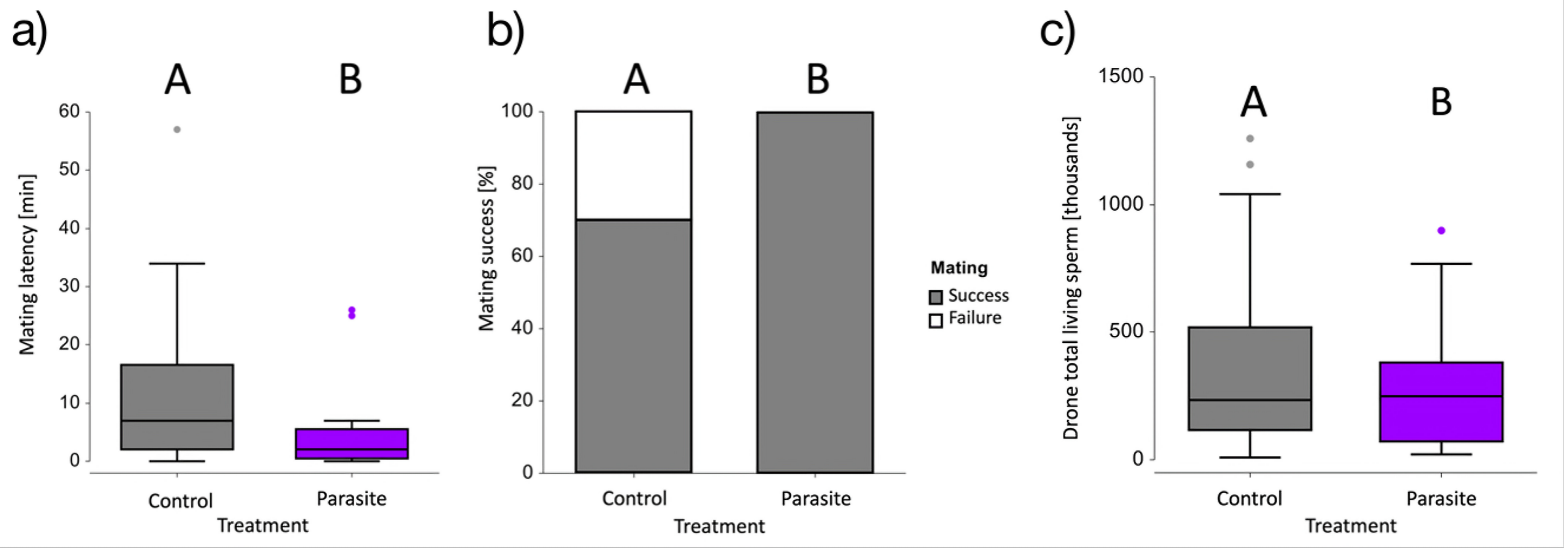
Mating latency, success and sperm quality of male (drone) bumblebees, *Bombus terrestris*, exposed to *Nosema ceranae*. (*a*) The mating latency in minutes of control (in grey) and exposed (in purple) drones. Exposed drones-initiated mating significantly faster than the controls (*p* < 0.05). (*b*) Stacked bar plot of mating success in the first round, successful mating is coloured in grey, while failure to mate is coloured white. Parasite exposed drones had a significantly increased mating success (*p* < 0.05). (*c*) Parasite exposed drones had significantly fewer total living sperm than control drones. In both boxplots the median, minimum, maximum and the first and third quartiles are displayed. Different capitalized letters (A, B) represent significant differences.

Parasite-exposed drones (5.69 ± 2.50) revealed a significantly shorter mating latency compared with the control drones (12.18 ± 2.93; mean [min] ± s.e.) (*z* = 6.09, *p* < 0.001; figure 4a), wherein exposed drone latency was 53% faster compared with controls. Likewise, parasite exposure had a significant positive effect on the mating success (Fisher’s exact test, *p* < 0.05; figure 4b), where success rates were 100 and 70% for parasite-exposed and control drones, respectively. When drones from the control treatment were presented a second gyne, 71% of the unsuccessful control drones were able to successfully mate. This yielded in an overall success rate in control drones of 91%. In contrast, parasite exposure revealed no significant effect on mating duration (*z* = 1.03, *p* = 0.30). Start mass of drones significantly, negatively correlated with mating duration (*z* = − 3.01, *p* < 0.01; electronic supplementary material, figure S4).

### 3.5. Drone total living sperm

Regardless of the treatment, drones (1.29 × 10^5^ ± 1.52× 10^3^) had a significantly lower number of total living sperm (*z* = − 4.43, *p* < 0.001) post-mating, when compared with unmated drones (3.12 × 10^5^ ± 2.803 × 10^4^; mean [total living sperm bee^−1^] ± s.e.), reflecting a 2.4-fold decrease. Furthermore, parasite exposure yielded a significant negative effect on total living sperm (*z* = − 2.19, *p* = 0.029; figure 4c), where exposed drones (2.76 × 10^5^ ± 3.13 × 10^4^) had 20% fewer total living sperm than control drones (3.43 × 10^5^ ± 4.43 × 10^4^; mean [total living sperm bee^−1^] ± s.e.). Considering infected individuals only, elevated spore counts lead to a significant reduction in total living sperm (*z* = − 2.46, *p* = 0.014).

## 4. Discussion

The data revealed that the microsporidian parasite *Nosema ceranae* can negatively affect traits of bumblebee workers and drones essential for colony fitness. In contrast to the workers, drones revealed an increased parasite infection rate and spore titres. In addition, infected drones revealed an increased mortality rate compared with their respective controls, which was not the case for workers. Furthermore, *N. ceranae* had a distinct negative effect on worker feeding glands which could result in inadequate brood care and impaired colony development [9]. Finally, parasite infections altered mating behaviour and reduced total living sperm in drones. Given the importance of these worker and drone traits to the inclusive fitness of bumblebee colonies [14], our data provide novel mechanistic explanations for how *N. ceranae* spillover may contribute to the declines in wild bumblebee populations. Our data highlight the urgent need to find effective, affordable and sustainable solutions to reduce the likelihood of inadvertent cross-species pathogen spillovers, to mitigate the effects and safeguard wild insect pollinators.

Regardless of treatment, and in line with previous findings (e.g. [14]), our data revealed sex-specific food consumption behaviour where workers consumed more than drones. Interestingly, while only based on the sucrose consumption, differences in consumption only become apparent after day 4—suggesting an underlying time-dependent factor. Differences in sucrose consumption are likely attributed to smaller workers typically having a higher basal metabolic rate [48] and less capacities to store energy in the form of fat [49], thus requiring higher food intake. In addition, higher nutritional requirements in workers compared with drones may be due to differences in physiology [50] and life-history traits (e.g. division of labour [51]). Workers for instance are known to continue developing essential tissues and organs, as well as proteinaceous-rich secretions (e.g. digestive enzymes produced in their feeding glands) well beyond the first 10 days post-eclosion [9]; this probably warrants increased nutritional demands [52]. In contrast, drone physiology is completed upon sexual maturity (i.e. roughly 4 days post-eclosion [44]), whereafter they typically leave their natal colonies and have a nectar-biased diet. Regardless of the underlying reasons, the consumption data must be interpreted with caution, as nutritional requirements may differ under field conditions [53]. Therefore, additional comparative data between sexes and castes (i.e. queens and workers) are needed to better understand how nutritional demands can vary. Such knowledge is critical to broaden our understanding of how diets may influence the impact of environmental stressors such as parasites on bumblebees.

In contrast to previous studies (e.g. [54]), our data provide no evidence for increased host consumption in response to energetic stress exerted by *N. ceranae*. Intriguingly, while only observed in drones, *N. ceranae* led to reduced pollen consumption. Whether the altered drone feeding behaviour reflects an adaptive response to parasite infection or host manipulation remains unclear. Indeed, several studies suggest that infected hosts can adapt their feeding behaviour in response to parasitism [55], whereas others show that parasites can alter the behaviour of their host to their own benefit [56]. Such conflicts between optimal diets for hosts and parasites emphasise the importance of how nutritional demands and subsequent feeding behaviours play a key role in understanding complex host–parasite interactions. Ultimately, the reduced pollen intake of the drones most certainly resulted in a deficiency in specific macro- or micro-nutrients that are essential for immune status and resistance to infections.

Our data reveal novel evidence that *N. ceranae* inoculations can successfully infect bumblebees when provided with a natural pollen diet—evident by the 2- and 53-fold increase in spores detected in gut samples of workers and drones, respectively, compared with the initially fed spore load. Furthermore, the observed infection rate for workers (26%) and drones (61%) lies well beyond those previously determined [30,57]. Moreover, our data suggest that drones were more susceptible to the parasite, evident by the elevated infection rates, spore titres and mortality. The observed differences in *N. ceranae* susceptibility between the sexes could be associated with the following factors: First, the nutritional status of the host significantly influences host–parasite dynamics, and the increased susceptibility of drones may be attributed to their altered pollen diet. Indeed, studies have shown that nutrient deficiencies can compromise the ability of bees to produce antimicrobial peptides, maintain a balanced gut microbiota, as well as detoxify, which are all essential for parasite defence [58,59]. The reduced pollen consumption observed in the drones may have facilitated the abilities of the parasite to replicate, strengthen the notion that the parasite may have manipulated the hosts consumption behaviour in its own favour [59]. Beyond varying diets, the increased susceptibility in drones may arise from sex-specific genetics affecting immunocompetence. Several past studies have indeed revealed gender differences in insect immune function, evident by males showing an overall reduced immunocompetence [60,61], such as reduced phagocytosis and lower activity levels of phenoloxidase. In the Hymenoptera, males are usually haploid whereas females are diploid, and heterozygosity has been argued to improve resistance to diseases [62]. Therefore, the observed increased susceptibility of drones to *N. ceranae* may be linked to hemizygosity at immune loci as heterozygosity likely enhances tolerance towards parasite infections [33]. Indeed, past studies have shown similar evidence supporting the haploid-susceptibility theory in bumblebees [63,64], honeybees [25,45], as well as in ants [65].

Regardless of whether the observed increased susceptibility in drones was due to a varying diet, haploid susceptibility or differences in life histories, the immune response is a costly trait that was most certainly triggered in both sexes by *N. ceranae* [66]. Subsequently, it is expected that there are trade-offs involved in the allocation of resources to the immune system that may have come at the expense of other key physiological traits [65]. Indeed, our data show that *N. ceranae* can impair HPG acini development in *B. terrestris* workers. While insecticides and malnutrition have also been shown to impair HPGs of bumblebees [14,16], this is the first study to demonstrate such an effect by a parasite. A similar reduction in HPG size has been observed in *N. ceranae* infected honeybees [67] and may be due to workers having less energetic resources available to adequately develop their HPGs or the parasite directly disrupting cellular development [68]. Whether this observed physiological response translates to impaired nursing behaviour or precocious shifts from nursing to foraging behaviour as observed when exposed to pesticides [15] remains to be tested. However, should the observed sublethal effects on HPGs impair collaborative brood care in bumblebees, this could result in severe consequences for colony development and the production of sexuals—and ultimately have negative effects for colony fitness and wild populations.

While *N. ceranae* has been shown to alter learning capacities and consumption behaviour in both bumblebees and honeybees [59,69], our data suggest that *N. ceranae* can alter male mating behaviour, causing an increased mating success rate. Parasites are known to affect their hosts mating behaviour as observed in numerous invertebrate species, including beetles [70], moths [71] and vertebrates such as rats [72]. At the mechanistic level, the observed increased mating success may be due to the parasite directly affecting the neuronal system of its host or the levels of relevant hormones [73]. Alternatively, the parasite may also be manipulating the pheromones or cuticular hydrocarbons of drones making them more attractive to females [74,75]. Considering that *Nosema* is known to be sexually transmitted [32], it appears apparent that *N. ceranae* would gain fitness benefits from enhancing its host’s sexual promiscuity, frequency of mating or duration of sexually active phases [73]. Indeed, our data suggest sexual transmission may play a significant role in the epidemiology of the parasite; as gynes were apparently not able to distinguish between infected and non-infected males, and mating success of *N. ceranae* infected drones was significantly higher than controls. However, due to the limited sample size the current findings are not sufficient to adequately answer this question, calling for additional mate choice experiments. While the faecal-oral route is undoubtedly the main pathway for horizontal transmission [76,77], data on the occurrence and efficiency of sexually transmitted *Nosema* spp. infections in wild bee populations remain to be determined. Additional field studies examining the prevalence of *Nosema* spp. infections in male bee gut and semen samples could offer insights on the risk of sexual transmission. In particular, determining the presence of spores in semen samples is critical, as male bees may be capable of eliminating *Nosema* spores from their seminal fluid [76], thereby reducing the risk of sexual transmission to females [77]. Irrespective of the potential risk of sexual transmission, mating with a *N. ceranae* infected male poses an additional risk as their reproductive physiology was impaired by the parasite.

Infections with *N. bombi* in bumblebees and *N. apis* in honeybees have been shown to impair sperm counts and sperm viability [32,78], respectively. Our novel results show that *N. ceranae* exposure can negatively affect sperm traits in bumblebees. Reasons for the observed negative effects on sperm viability may be attributed to the parasite causing oxidative stress and/or reducing seminal fluid protein abundance [79]. As spermatogenesis and spermiogenesis are completed upon emergence in bees, the reduced sperm quantity suggests that parasite infections may interfere with the migration of sperm from the testis to the seminal vesicles. Furthermore, considering parasitized males consumed less pollen, it appears plausible that a lack of critical nutrients (e.g. proteins or macro- and micro-nutrients) may have negatively affect the development and/or functioning of genitalia muscular tissues [16], thereby explaining the reduced sperm quantity. Our data do not enable to untangle whether the parasite or malnutrition alone was responsible for the effect, or if even a potential interactive effect was at hand. Nevertheless, considering males bees of the genus *Bombus* can mate multiple females, any reduction in sperm quality and in particular sperm quantity constitutes a clear fitness constraint. Assuming *B. terrestris* gynes store on average 40 000 sperm in their spermatheca [80], the control males would have had sufficient sperm to inseminate at least nine gynes. Parasitized males in contrary would only have been able to inseminate a total of six gynes, resulting in a reduction in male fitness of 33%. Furthermore, reduced sperm viability may reduce the probability of producing viable worker eggs or having a male-biased offspring sex ratio, which would reduce female worker force and disrupt colony development. However, the overall impact of impaired sperm quality remains to be tested. Indeed, reduced sperm quantity and quality may not necessarily imply any constraints at the colony level, as female bumblebees store excess sperm in their spermatheca [80] and only a small proportion of the stored sperm is ultimately required to complete a successful colony cycle.

In summary, the data provide novel evidence that *N. ceranae* infections can impair key traits of bumblebee’s colony fitness (i.e. collaborative brood care and functionality of male sexuals). Since parasitized workers had smaller HPG acini, and acini size is a robust proxy for their functionality [81], it is plausible that their adequate functionality was impaired. Consequently, infected workers may process less and/or lower quality food which could compromise their ability to adequately feed themselves and provision brood. Furthermore, the reduced sperm traits mirror a potential drastic scenario for bumblebee male fitness. While additional studies are required to fully understand what factors aid a successful infection of *N. ceranae* in bumblebees, it appears evident that the parasite is indeed an emerging infectious agent and may contribute to recent bumblebee population declines [28,29].

## Data Availability

The complete raw data can be found at the Dryad repository [82]. Supplementary material is available online [83].
